# Endothelial progenitor cells and plaque burden in stented coronary artery segments: an optical coherence tomography study six months after elective PCI

**DOI:** 10.1186/s12872-017-0534-1

**Published:** 2017-04-26

**Authors:** Sylvia Otto, Kristina Nitsche, Christian Jung, Aleh Kryvanos, Andrey Zhylka, Kerstin Heitkamp, Juan-Luis Gutiérrez-Chico, Björn Goebel, P. Christian Schulze, Hans R. Figulla, Tudor C. Poerner

**Affiliations:** 1 Department of Internal Medicine I, Division of Cardiology, Angiology, Pneumology and Intensive Medical Care, University Hospital Jena Friedrich-Schiller-University Jena, Am Klinikum 1, 07747 Jena, Germany; 2 Department of Cardiology, Pulmonology, University, Duesseldorf, Medical Faculty and Vascular Diseases, Düsseldorf, Germany; 3 UltraOsteon, Mannheim, Germany; 4 Belarusian State University, Faculty of Applied Mathematics and Computer Science, Minsk, Belarus; 5 Cardiology Department, Krankenhaus Frankfurt/Oder, Frankfurt/Oder, Germany

**Keywords:** Endothelial progenitor cells, PCI, Optical coherence tomography, atherosclerosis, Coronary plaque

## Abstract

**Background:**

Endothelial progenitor cells (EPC) are involved in neovascularization and endothelial integrity. They might be protective in atherosclerosis. Optical coherence tomography (OCT) is a precise intracoronary imaging modality that allows assessment of subintimal plaque development. We evaluated the influence of EPC on coronary plaque burden in stable disease and implemented a novel computational plaque analysis algorithm using OCT.

**Methods:**

Forty-three patients (69.8% males, 69.6 ± 7.7 years) were investigated by OCT during re-angiography 6 months after elective stent implantation. Different subpopulations of EPCs were identified by flow cytometry according to their co-expression of antigens (CD34+, CD133+, kinase domain receptor, KDR+). An algorithm was applied to calculate the underlying total plaque burden of the stented segments from OCT images. Plaque morphology was assessed according to international consensus in OCT imaging.

**Results:**

A cumulative sub-strut plaque volume of 10.87 ± 12.7 mm^3^ and a sub-stent plaque area of 16.23 ± 17.0 mm^2^ were found within the stented vessel segments with no significant differences between different stent types. All EPC subpopulations (mean of EPC levels: CD34+/CD133+: 2.66 ± 2.0%; CD34+/KDR+: 7.50 ± 5.0%; CD34+/CD133+/KDR+: 1.12 ± 1.0%) inversely correlated with the identified underlying total plaque volume and plaque area (*p* ≤ 0.012).

**Conclusions:**

This novel analysis algorithm allows for the first time comprehensive quantification of coronary plaque burden by OCT and illustration as spread out vessel charts. Increased EPC levels are associated with less sub-stent coronary plaque burden which adds to previous findings of their protective role in atherosclerosis.

## Background

Atherosclerosis is a systemic disease. Our understanding of its underlying pathomechanism has been extended beyond typically contributing cardiovascular risk factors like hyperlipidemia, hypertension, diabetes mellitus or nicotine abuse. Lately, individual biological factors such as endothelial progenitor cells (EPC) are in the focus of translational research. EPC are pluripotent, mononuclear cells deriving from bone marrow. They are found to be involved in new vessel formation and vascular regeneration after arterial injury [1–4]. There is growing evidence that some EPC subpopulations, characterized by surface markers like CD34, CD133 or KDR, might exert protective properties in atherosclerosis [5–8]. Common cardiovascular risk factors like smoking, obesity, hypercholesterolemia, diabetes, inactivity or chronic inflammation are associated with reduced numbers and impaired function of EPC [9–12]. Whereas statins, ACE inhibitors, omega-3 fatty acids, estrogens and high-density lipoprotein among others have been correlated with increased EPC levels [13, 14].

Optical coherence tomography (OCT) has emerged as the new gold-standard of intracoronary imaging due to its high resolution, fast acquisition and ease of handling in the catheterization laboratory [15, 16]. Consensus in interpreting OCT images regarding its various aspects (e.g. vessel wall, stent struts, scaffold boxes, thrombi, etc.) has been established [17–19]. However, standardized algorithm to analyse OCT pullbacks and to enhance comparability of results are still lacking [20]. Coronary plaques, their relation to disease progression and to acute coronary syndromes (“vulnerable plaques”) as well as to individual biomarkers are of increasing interest. Therefore and beyond a thorough analysis of atherosclerotic plaques, quantification of coronary plaque burden is needed.

## Objectives

We (1) assessed the influence of EPC on coronary plaque burden using OCT in stented vessel segments in stable coronary artery disease (CAD). (2) A novel parameter of plaque burden “luminal surface area” was introduced and a computational OCT algorithm was implemented that allows quantification of coronary plaques in a segment of interest and subsequent visualization as comprehensive spread-out vessel charts.

## Methods

### Study design and patient population

The study was conducted according to the principles of the Declaration of Helsinki and approved by the local ethics committee of the University Hospital of Jena. The main study (OCTOPUS trial) is registered at clinicaltrials.gov, NCT:01056744. All study participants gave written informed consent. The data presented in this manuscript are collected in a sub-study from the OCTOPUS trial. The design and results of the main study have already been published [21, 22]. Briefly, the trial was intended to randomly compare two interventional devices (Xience V™ drug-eluting stents vs. bare metal stents post-dilated with a paclitaxel-coated balloon) in patients with stable CAD.

### Optical coherence tomography and identification of coronary plaques

Time-domain OCT imaging (M2 CV system, LightLab Imaging Inc., Westford, MA, USA) of the stented vessel segment (region of interest, ROI) was performed six months after the index percutaneous coronary intervention (PCI) according to the study protocol. Due to the old occlusive technique OCT imaging was limited to the stented vessel segment. OCT images were analyzed frame by frame.

**Fig. 1:**
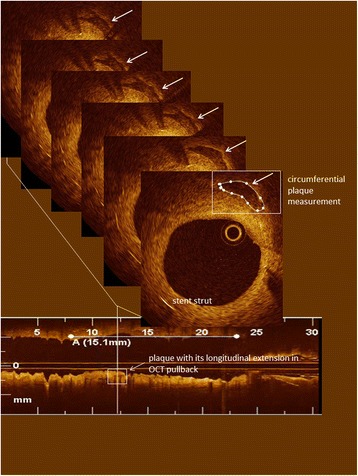
Example of a 30 mm OCT pullback (bottom) with an identified calcified plaque (vertical line). The plaque is followed cross-section per cross-section and corresponding circumferential plaque measurements are shown. Plaque volume was calculated by the integral of each 2-dimensional plaque measurement over identified plaque length. Horizontal line (A) shows length of stent within the pullback. Stent struts are seen at 3 and 7 o’clock in the cross-section

A novel algorithm was applied to calculate total plaque burden in terms of underlying total plaque volume and total luminal plaque surface area of a stented vessel segment: Each cross section was analyzed regarding the existence of atherosclerotic plaques situated abluminal from stent struts. Plaque morphology was assessed according to international consensus and each plaque was classified as fibrotic, fibrocalcific, fibroatheroma or mixed [17–19]. Subintimal/sub-strut plaques in the treated vessel segment were manually traced in each cross section and entered into a computational algorithm (Fig. 1).

Quantitative plaque assessment consisted of:

- Plaque count of each subtype
- Volumes of each plaque were computed through the integral of cross-sectional plaque measurements over plaque length (Fig. 1) according to the formula:

$$ {Vol}_{Plaque}={\int}_{x_d}^{x_p}{CSA}_{Plaque\;} dx $$

(Vol_Plaque_: plaque volume, x_p_: proximal plaque location, x_d_: distal plaque location, CSA_Plaque_: cross-sectional plaque area on each frame)

**Fig. 2:**
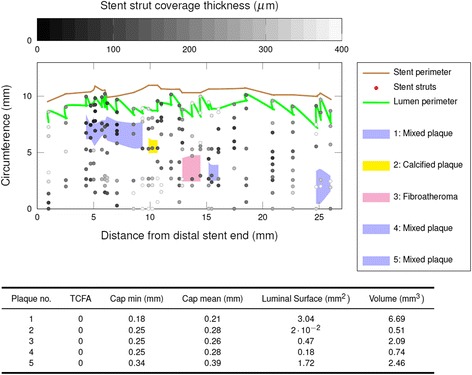
Schematic illustration of sub-strut plaque distribution in an analyzed coronary segment as spread-out vessel chart. The investigated vessel segment is displayed longitudinally bisected and unfolded: Stent struts are displayed as grey dots according to their degree of stent incorporation in the vessel wall (neointimal thickness: grey color bar). Polygones display luminal surface plaque areas of five different identified sub-stent plaques (pink = fibroatheroma, yellow = calcified plaques blue = mixed plaque). X-axis = stent length, y-axis = stent circumference. Corresponding measurements including cap thickness, “luminal” surface area and volume of each plaque are given in the table below

**Fig. 3:**
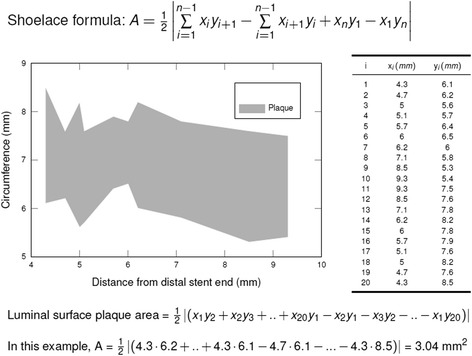
Shoelace formula. Schematic presentation of the mathematical algorithm using the Shoelace formula to calculate plaque luminal surface areas by consecutive measurements of 20 cross-sections as shown in the table on the right

- Luminal surface area of each plaque, as displayed in the spread-out vessel chart (Fig. 2 - top), was calculated using the Gauss’s area formula, also known as Shoelace formula. Fig. 3 describes this mathematical method to determine areas as simple polygons “projected” at the luminal surface for coherent visualization (Fig. 3) [23, 24].
- Minimal cap thickness, manually measured at its narrowest site of magnified OCT cross-sections, using the mean value of two measurements.

The changing position of the imaging catheter might have been misleading if only polar coordinates using its position had been used. Therefore, the centroid of the cross-section and not the imaging catheter was used as a reference for determination of coordinates.

Finally, total volume and total luminal surface area were computed for each morphologic plaque subtype (fibrotic, fibroatheroma, fibrocalcific and mixed) for the analysed vessel segment (Fig. 2 - bottom). In 7 patients measurements of plaque surface area and volume have been performed separately by two independent observers (K.H., K.N.) to assess variability of observations.

### Laboratory analysis of endothelial progenitor cells

Peripheral venous blood samples were drawn a day prior PCI for assessment of EPC additional to routine blood work according to the hospital’s standard operating procedures. EPC were identified by flow cytometry according to their surface antigens CD34, CD133 and KDR as described before [9, 25]. Red blood cells were lysed using lysing solution. Then, cells were incubated with an FcR blocking reagent (MACS Milteny Biotec) to saturate sites for unspecific binding and washed twice with phosphate-buffered saline (PBS). Peripheral monoclonal blood cells were stained with fluorescein-isothiocyanate (FITC)-conjugated anti-human CD34 monoclonal antibody (MACS Milteny Biotec, Clone AC136), R-phycoerythrin (R-PE) conjugated anti-human CD133 mononclonal antibody (MACS Milteny Biotec, Clone 293C3), and allophycocyanin (APC)-conjugated anti-human VEGFR-2 monoclonal antibody (R&D Systems, Clone 89,106) after incubating for 15 min at 4 °C. Isotype- and species-matched antibodies (mouse IgG2a FITC-conjugated antibodies and IgG2b PE-conjugated antibodies) were used as controls (MACS Milteny Biotec, Clone S43.10 and Clone IS6-11E5.11). In this study, three different EPC populations (CD34+/CD133+, CD34+/KDR+ and CD34+/CD133+/KDR+) were measured by 3-color flow cytometry (FACS Calibur; BD Biosciences) and analyzed (CellQuestPro, version 4.0.2 BD Biosciences).

### Statistical Analysis

All parameters were archived in a custom-made database (Microsoft Access, Microsoft Inc.). All calculations were done with SPSS (version 21.0, IBM SPSS statistics). Continuous variables are expressed as mean ± SD and categorical variables are presented as percent. Bivariate analysis with the Pearson correlation coefficient was used to determine the strength of relationship between EPC levels and plaque burden. Multiple linear regression analysis was done to test for potential confounding variables. The variability of plaque volume and surface area measurements has been assessed using 2-tailed paired Student’s t test and Bland and Altman agreement analysis between two independent observers blinded to each other’s results. Statistical significance was assumed for *p*-values <.05 (two-tailed).

## Results

### Study population and procedural characteristics

In this sub-study of the OCTOPUS trial 44 lesions in 43 patients were investigated with OCT during re-angiography 6 months after elective coronary stenting. Table 1 shows the clinical characteristics of the study population. Most patients were male (69.8%) and a high percentage of coronary risk factors were observed (Table 1). All patients received statin medication. Procedural characteristics of the study population are described in Table 2. Most stented lesions were rather complex (only AHA/ACC type B or C lesions; 20.5% chronic total occlusions, Table 2). All lesions were treated with a drug-eluting device according to the main study protocol: Drug-eluting stents (DES, Xience V™, Abbott Vascular, IL) were implanted in 19 lesions and bare metal stents (BMS, Coroflex Blue™, BBraun Melsungen, Germany) post-dilated with a paclitaxel-coated balloon (DCB, Sequent Please™, BBraun Melsungen, Germany) were used in 25 lesions. Invasive follow-up was conducted after 188.4 ± 20 days. Except for two lesions, all treated segments showed a good result (restenosis <20%) at 6-month follow-up (f/u). Significant restenosis of the target lesion was found in only two patients (1 BMS, 1 DES) with subsequent re-PCI during scheduled invasive f/u. Furthermore, in two patients (1 DES and 1 BMS), we found lesion development of a non-target vessel with indication for revascularization (PCI) at f/u [26]. No differences in EPC counts were found between the groups of DES vs. BMS/DCB.

**Table 1:** Baseline clinical characteristics and levels of endothelial progenitor cells of the study population

Characteristics	*n* = 43 patients
	mean ± SD or N (%)
Age	69.4 ± 7.5
Male gender	30 (69.8%)
Hypertension	43 (100%)
Diabetes mellitus type 2	18 (41.9%)
Hyperlipidemia	31 (72.1%)
Smoker/Ex-Smoker	15 (34.9%)
GFR (ml/min)	70.1 ± 27.4
LDL (mmol/l)	2.72 ± 1.4
Statin therapy	43 (100%)
EPC levels	
CD 34+/CD133+ (%)	2.66 ± 2.0
CD 34+/KDR+ (%)	7.45 ± 5.1
CD 34+/CD 133+/KDR+ (%)	1.11 ± 1.0

*LDL* low-density lipoprotein-cholesterol, *GFR* glomerular filtration rate, *EPC* endothelial progenitor cells, *SD* standard deviation. Subpopulations of endothelial progenitor cell (EPC) counts are presented as % of the relevant gate

**Table 2:** Procedural characteristics of the study population

Characteristics	*n* = 44 lesions
	mean ± SD or N (%)
BASELINE	
Reference lumen diameter (mm)	2.62 ± 0.34
Minimal lumen diameter (mm)	0.66 ± 0.4
Stenosis (%)	74.5 ±13.9
Stent type: BMS / DES	25 (56.8%) / 19 (43.2%)
Stent length (mm)	19. 8 ± 4.9
≥ 2 stents	5 (11.4%)
Stent diameter (mm)	2.82 ± 0.24
Ostial lesion	3 (6.8%)
Chronic total occlusion	9 (20.5%)
Lesion type (AHA/ACC) A/B/C	0 / 32 (72.7%) / 12 (27.3%)
Target vessel RCA/LCX/LAD	14 (31.8%) / 10 (22.7%) / 20 (45.5%)
Fluoroscopy time (min)	11.6 ± 10.5
FOLLOW-UP	
Follow-up interval (days)	188.4 ± 20.0
Minimal lumen diameter (mm)	2.1 ± 0.4
Diameter Stenosis (%)	21.1 ± 12.5

*BMS* bare metal stent, *DES* drug-eluting stent, *RCA* right coronary artery, *LCX* left circumflex, *LAD* left anterior descending, *SD* standard deviation

### Coronary Plaque Burden and Association with EPC

EPC levels were measured in all patients at baseline, and their mean values are displayed in Table 1. Leukocyte and lymphocyte numbers, which could bias EPC counts, ranged within standard normal values in all patients. A total of 110 coronary plaques were identified in 44 stented vessel segments (ROI) by OCT at 6-month invasive f/u. More precisely, coronary plaques were found in all, but 2 vessel segments. Table 3 shows the distribution of different plaque types per ROI. There was a mean of 2.5 ± 1.5 plaques per ROI (Table 3). Intracoronary thrombi within the treated vessel segment were not observed. Since we investigated stented vessel segments thin-cap fibroatheroma (TCFA) were not observed by definition. Focal tissue prolapse into the lumen between stent struts was found in five stents with a range of 1 to 5 different intra-stent localizations. Microvessels were only observed in one ROI. Overall, a total plaque volume of 7.5 ± 8.5 mm^3^ and a luminal plaque surface area of 12.4 ± 13.7 mm^2^ were found per ROI with no significant differences between different stent types (Table 3). Of note, we did not find any neoatherosclerotic plaques within neointima tissue formation.

**Table 3:** OCT analysis of coronary plaque burden in each stented vessel segment (ROI)

Plaque classification	N (mean ± SD)/ ROI	N (range)/ROI	Volume (mm^3^)	Surface Area (mm^2^)
Pathologic intimal thickening	0.1 ± 0.3	0–1	1.0 ± 3.5	3.2 ± 10.7
Fibrotic plaque	0.2 ± 0.5	0–2	0.5 ± 1.6	1.0 ± 3.1
Fibrocalcific plaque	0.2 ± 0.4	0–2	0.4 ± 1.2	0.8 ± 2.6
Fibroatheroma	0.8 ± 0.8	0–3	3.4 ± 5.8	4.7 ± 6.7
Mixed Plaques	0.6 ± 1.0	0–4	2.3 ± 5.3	2.7 ± 5.8
Total Plaques	2.5 ± 1.5	0–6	7.5 ± 8.5	12.4 ± 13.7

*ROI* region of interest, *N* number, *SD* standard deviation

**Fig. 4:**
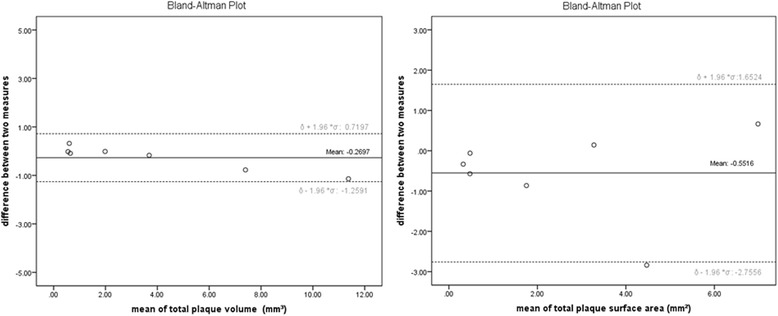
Bland-Altman plots. Interobserver agreement for plaque volume (left) and plaque luminal surface area (right) measurements

Variability of observations has been assessed on two independent sets of measurements of total plaque volume and total plaque surface area within the stented segment. The mean absolute differences and their standard deviations were: 0.27 ± 0.51 mm^3^ and 0.55 ± 1.12 mm^2^, respectively. Corresponding Bland and Altman plots are given in Fig. 4. No significant differences were found between independent measurements of each analyzed plaque with respect to surface area and volume.

**Fig. 5:**
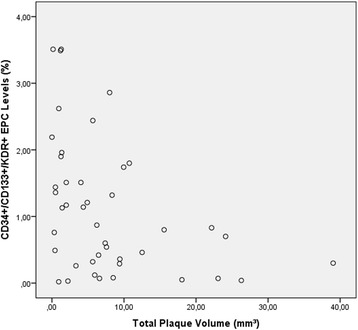
Scatter plot. Negative correlation between EPC levels (y-axis) and total plaque volume (x-axis)

All three EPC subpopulations inversely correlated with the identified sub-stent plaque volume and luminal plaque surface area (Fig. 5, Table 4) showing a significance of *p* ≤ 0.012. No associations were found between coronary plaque burden and various proliferation parameters (angiography and OCT) as well as stent apposition and stent strut coverage. Multivariable analysis could exclude parameters like LDL-cholesterol, age and creatinine as confounders of the correlation between EPC and plaque burden (Table 5). Using a univariate analysis in a general linear model including also the treatment arm no further significant associations were found.

**Table 4:** Association between coronary plaque burden and EPC levels

EPCs	Total Plaque Volume	*p*	Plaque Surface Area	*p*
CD 34+/CD133+	*r* = − 0.402	0.007	*r* = − 0.410	0.006
CD 34+/KDR+	*r* = − 0.390	0.010	*r* = − 0.380	0.012
CD 34+/CD 133+/KDR+	*r* = − 0.409	0.006	*r* = − 0.388	0.010

*R* correlation coefficient, *EPC* endothelial progenitor cells

**Table 5:** Multiple linear regression model (*R* ^2^ 0.235, F 2.675, *p* = 0.048) for EPC and possible covariates for coronary plaque burden (total plaque volume): similar significant results were found for the other investigated EPC subpopulations and for plaque surface area

Risk factor	Regression coefficient *B*	Standard error	*p*	95% CI for *B*
(Constant)	25.51	13.86	*.041*	1.35; 57.67
Age	−0.19	0.17	*.499*	−0.47; 0.23
LDL	-1.16	1.02	*.265*	−3.21; 0.91
Creatinine	−0.04	0.03	*.180*	−0.95; 0.18
CD34+/CD133 + EPC	-2.43	0.78	***.004***	−4.0; −0.85

*LDL* low-density lipoprotein-cholesterol, *GFR* glomerular filtration rate, *EPC*, endothelial progenitor cells, *CI* confidence interval

### Visualization of OCT findings as spread out vessel charts

To visualize OCT findings, spread out vessel charts of each stented vessel segment (ROI) were generated using the computational algorithm. Vessel segments are displayed longitudinally, unfolded and bisected in these spread outs, and give an en-face view of atherosclerotic plaques and their spatial relation to adjacent structures like overlaying stent struts or side branches (Fig. 2). Color-coding was added to identify lumen and stent perimeter, plaque type and stent coverage (neointimal thickness in grey color bar) at a glance (Fig. 2).

## Discussion

In this study, we investigated atherosclerotic plaque burden of treated vessel segments by OCT six-months after elective PCI. To the best of our knowledge, this is the first study analyzing OCT-derived coronary plaque measurements and their association to individual EPC levels.

We found a mean of 2.5 ± 1.5 plaques with a volume of 7.5 ± 8.5 mm^3^ per stented vessel segment. Most plaques were fibroatheroma or mixed, lipid-enriched plaques. Neoatherosclerotic plaque formation was not observed six months after stent implantation.

EPC are seen as a surrogate biomarker of cardiovascular health and vascular functional status [7]. A decreased EPC pool has been linked to adverse cardiovascular events, in-stent neointimal hyperplasia and endothelial dysfunction [5–7, 25]. Current evidence for the protective role of EPC mainly derives from animal and ex vivo studies, while most clinical studies rely on surrogate parameters for atherosclerotic disease severity [27–30]. So far, human EPC studies measuring atherosclerotic plaques directly have not been reported except for studies investigating carotid-intima media thickness [31–33]. Our data show a strong inverse association between the numbers of circulating EPC and coronary plaque burden measured as plaque volume and plaque surface area by OCT in a clinical study of patients with stable CAD and previous PCI.

Evidence regarding the role of EPC in atherogenesis has been somewhat conflicting. Their protective effects were doubted, and even pro-atherogenetic properties have been postulated in the past [34–36]. A few fundamental questions have to be raised in this controversy: (1) Which phenotype of EPC was investigated? (2) How is their functional status? (3) How do they execute their effects and which mediators and mechanism trigger their action? Unfortunately, there is no exclusive EPC marker. Despite a large number of in vitro and in vivo studies, it is still methodologically challenging and remains unclear which discrete population of EPC really triggers adult vasculogenesis and exerts anti-atherosclerotic effects. Currently, the combined expression of CD34+, CD133+ and KDR+, are considered as the definition for EPC [9, 37]. Our study, investigating this predefined EPC phenotype shows that a high EPC count is associated with only marginal coronary plaque burden. However, we cannot draw any causal relation with the findings of this study. A larger plaque might be related to a lower number of regenerative EPC as well as a lower number of EPC might be caused by higher consumption and exhaustion of the bone marrow due to general atherosclerotic disease.

Recently, EPC have been incorporated as a key therapeutic target in a novel stent design for interventional treatment of coronary stenoses: The Combo™ stent (OrbusNeich Medical) is a drug-eluting stent consisting of sirolimus coating on the abluminal stent side and EPC capturing antibodies on the luminal stent side [38, 39]. IVUS imaging revealed a relevant difference regarding formation of neoatherosclerosis. EPC capturing led to a more homogenous fibrotic in-stent neointima without signs of a confluent necrotic core, which adds to previous EPC studies on plaque stabilization by modifying plaque composition, and thus reducing plaque vulnerability. [28, 38] However, this could not translate into a measurable clinical benefit for the treated patients. Our study discovered the link between EPC count and plaque burden for the first time. In our study we only provide plaque burden of stented vessel segments at a predefined time point. Our data do not allow conclusion on plaque stabilization since we did not perform OCT at baseline. Further studies are needed to longitudinally evaluate possible alterations in plaque burden and composition with respect to variations of EPC counts and systemic or local therapeutic interventions (e.g. drug-eluting devices, statins, anti-diabetic medication). Due to the relatively small sample size we are not able to further evaluate possible interactions of the used antiproliferative drug (everolimus vs. paclitaxel), duration of drug action (polymer coating vs. single shot application by a balloon) and individual biological factors like EPCs. Unfortuantely previous interventional studies investigating EPC counts and outcomes did not state the used types of stents [8, 40]. Also, we cannot anticipate the strength of correlation between the plaque content of a stented vessel segment and an entire native coronary vessel in stable CAD.

Currently, the PREMIER trial is investigating a strategy of intense lipid elimination (statin therapy + LDL-apheresis) on atheroma volume reduction and EPC mobilization compared to standard statin therapy [41]. A bias regarding co-medication in our study is unlikely since all patients received statin therapy.

### Methodology of coronary plaque assessment

Plaque volume assessment has been mainly conducted with IVUS. OCT studies have been focusing on TCFAs and ruptured plaques in ACS patients. [42–47]. Less sophisticated OCT plaque analysis using just the count of observed plaques or plaque circumference measurements (e.g. lipid arc) are insufficient to estimate the entire plaque burden and severity of atherosclerosis. Plaque length was for example only measured in areas with a lipid arc >90°, which also is a somewhat random cut-off. [48] Moreover, lipid arc measurements are prone to significant errors due to the variable eccentric position of the imaging catheter in the vessel. Contrary, our algorithm allows a more detailed quantification of plaque burden in an area or vessel of interest, including all noticeable plaques irrespective of size. We also introduce a novel parameter - luminal surface area of plaques – which we see as a more reproducible measure of plaque burden, since it describes and measures for the first time the projection of a plaque on the luminal surface. Given the relevance of TCFAs it might be of interest if this new parameter of plaque dimension is better suited to predict adverse events (e.g. TCFA with larger luminal surface area) than “classic” plaque size parameters (depth, length, volume). Moreover, we report a computational algorithm that enables in vivo quantification of the atheroma in a region of interest. Our 2D reconstruction of the luminal surface of a vascular segment superimposes information on plaque localization and extension, coverage thickness of struts in addition to strut localization, as well as stent und lumen perimeter (Fig. 2). Nevertheless, further longitudinal studies and especially a consensus about standardized plaque evaluation are needed to help its implementation into clinical practice.

### Clinical implications

Taken the current evidence and the results of this study into account, EPC are an interesting specific target for prevention and disease modification of atherosclerosis. Furthermore, a low EPC count seems to be an independent risk factor for coronary atheroma burden. Translational research including in vivo imaging is needed to identify the link between EPCs, chemokines and atherosclerotic plaque development.

### Limitations

Measurements of lipid-rich plaques are limited due to axial resolution of OCT. Therefore, precise definition of plaque borders far distally from the lumen can be challenging and partly insufficient. While the volumetric assessment of fibroatheroma might be therefore difficult, the surface area measurements of all plaques should not be hampered by this methodological drawback. Our data are stemming from a subgroup of the OCTOPUS trial with only moderate sample size (*n* = 44), and therefore the correlation coefficients are relatively low. Due to the occlusive OCT-technique with considerably shorter pullbacks compared to the newer generation OCT, plaque analysis was limited to the stented vessel segment. Since OCT was not performed at baseline, sequential data for coronary plaque burden cannot be provided. Taken these limitations together, our study results are rather descriptive and hypothesis generating, and should be followed by sequential plaque analysis studies in native coronary vessels. Finally, we cannot preclude on potential therapy-related atherosclerotic changes of stented plaques.

## Conclusion

Our computational OCT analysis algorithm is feasible, allows quantification of coronary atheroma burden and comprehensible illustration as spread out vessel charts. Increased EPC levels are associated with less coronary plaque burden of a stented vessel segment, which adds to previous findings of their protective role in atherosclerosis. Further research is needed to refine EPC as a possible therapeutic target and thereby address individual biological differences.

## Abbreviations

BMS: Bare metal stents
CAD: Coronary artery disease
CI: Confidence interval
DCB: Paclitaxel-coated balloon
DES: Drug-eluting stent
EPC: Endothelial progenitor cells
f/u: Follow-up
GFR: Glomerular filtration rate
KDR: Kinase domain receptor
LAD: Left anterior descending artery
LCX: Left circumflex artery
LDL: Low-density lipoprotein
OCT: Optical coherence tomography
PCI: Percutaneous coronary intervention
RCA: Right coronary artery
ROI: Region of interest
SD: Standard deviation
TCFA: Thin-cap fibroatheroma

## References

[1] Walter DH, Dimmeler S (2002). Endothelial progenitor cells: regulation and contribution to adult neovascularization. Herz.

[2] Kong D, Melo LG, Gnecchi M, Zhang L, Mostoslavsky G, Liew CC, Pratt RE, Dzau VJ (2004). Cytokine-induced mobilization of circulating endothelial progenitor cells enhances repair of injured arteries. Circulation.

[3] Werner N, Junk S, Laufs U, Link A, Walenta K, Bohm M, Nickenig G (2003). Intravenous transfusion of endothelial progenitor cells reduces neointima formation after vascular injury. Circ Res.

[4] Asahara T, Masuda H, Takahashi T, Kalka C, Pastore C, Silver M, Kearne M, Magner M, Isner JM (1999). Bone marrow origin of endothelial progenitor cells responsible for postnatal vasculogenesis in physiological and pathological neovascularization. Circ Res.

[5] Werner N, Kosiol S, Schiegl T, Ahlers P, Walenta K, Link A, Bohm M, Nickenig G (2005). Circulating endothelial progenitor cells and cardiovascular outcomes. N Engl J Med.

[6] Schmidt-Lucke C, Rossig L, Fichtlscherer S, Vasa M, Britten M, Kamper U, Dimmeler S, Zeiher AM (2005). Reduced number of circulating endothelial progenitor cells predicts future cardiovascular events: proof of concept for the clinical importance of endogenous vascular repair. Circulation.

[7] Hill JM, Zalos G, Halcox JP, Schenke WH, Waclawiw MA, Quyyumi AA, Finkel T (2003). Circulating endothelial progenitor cells, vascular function, and cardiovascular risk. N Engl J Med.

[8] George J, Herz I, Goldstein E, Abashidze S, Deutch V, Finkelstein A, Michowitz Y, Miller H, Keren G (2003). Number and adhesive properties of circulating endothelial progenitor cells in patients with in-stent restenosis. Arterioscler Thromb Vasc Biol.

[9] Jung C, Rafnsson A, Shemyakin A, Bohm F, Pernow J. Different subpopulations of endothelial progenitor cells and circulating apoptotic progenitor cells in patients with vascular disease and diabetes. Int J Cardiol. 2010;143(3):368–72. doi:10.1016/j.ijcard.2009.03.075. 10.1016/j.ijcard.2009.03.075 19398138

[10] Laufs U, Wassmann S, Czech T, Munzel T, Eisenhauer M, Bohm M, Nickenig G (2005). Physical inactivity increases oxidative stress, endothelial dysfunction, and atherosclerosis. Arterioscler Thromb Vasc Biol.

[11] Chen JZ, Zhang FR, Tao QM, Wang XX, Zhu JH, Zhu JH (2004). Number and activity of endothelial progenitor cells from peripheral blood in patients with hypercholesterolaemia. Clin Sci (Lond).

[12] Wang X, Zhu J, Chen J, Shang Y (2004). Effects of nicotine on the number and activity of circulating endothelial progenitor cells. J Clin Pharmacol.

[13] Zhu JH, Tao QM, Chen JZ, Wang XX, Zhu JH, Shang YP (2004). Statins contribute to enhancement of the number and the function of endothelial progenitor cells from peripheral blood. Sheng Li Xue Bao.

[14] Pellegatta F, Bragheri M, Grigore L, Raselli S, Maggi FM, Brambilla C, Reduzzi A, Pirillo A, Norata GD, Catapano AL (2006). In vitro isolation of circulating endothelial progenitor cells is related to the high density lipoprotein plasma levels. Int J Mol Med.

[15] Iannaccone M, D'Ascenzo F, Templin C, Omede P, Montefusco A, Guagliumi G, Serruys PW, Di Mario C, Kochman J, Quadri G, et al. Optical coherence tomography evaluation of intermediate-term healing of different stent types: systemic review and meta-analysis. Eur Heart J Cardiovasc Imaging. 2017;18(2):159–66. doi:10.1093/ehjci/jew070. 10.1093/ehjci/jew070 27099274

[16] Iannaccone M, Quadri G, Taha S, D'Ascenzo F, Montefusco A, Omede P, Jang IK, Niccoli G, Souteyrand G, Yundai C (2016). Prevalence and predictors of culprit plaque rupture at OCT in patients with coronary artery disease: a meta-analysis. Eur Heart J Cardiovasc Imaging.

[17] Prati F, Regar E, Mintz GS, Arbustini E, Di Mario C, Jang IK, Akasaka T, Costa M, Guagliumi G, Grube E (2010). Expert review document on methodology, terminology, and clinical applications of optical coherence tomography: physical principles, methodology of image acquisition, and clinical application for assessment of coronary arteries and atherosclerosis. Eur Heart J.

[18] Prati F, Guagliumi G, Mintz GS, Costa M, Regar E, Akasaka T, Barlis P, Tearney GJ, Jang IK, Arbustini E (2012). Expert review document part 2: methodology, terminology and clinical applications of optical coherence tomography for the assessment of interventional procedures. Eur Heart J.

[19] Tearney GJ, Regar E, Akasaka T, Adriaenssens T, Barlis P, Bezerra HG, Bouma B, Bruining N, Cho JM, Chowdhary S (2012). Consensus standards for acquisition, measurement, and reporting of intravascular optical coherence tomography studies: a report from the International Working Group for Intravascular Optical Coherence Tomography Standardization and Validation. J Am Coll Cardiol.

[20] Otto S, Gassdorf J, Nitsche K, Gutiérrez-Chico J, Kryvanos A, Goebel B, Schulze PC, Figulla HR, Poerner TC. Time Course of Vascular Response after an A Priori Strategy of Bare Metal Stent Implantation Post-Dilated with a Paclitaxel-coated Balloon: Implementation of a Three Dimensional Analysis Algorithm with Optical Coherence Tomography. Cardiol J. 2016;23(3):296–306. doi:10.5603/CJ.a2016.0018. 10.5603/CJ.a2016.0018 27064798

[21] Poerner TC, Otto S, Gassdorf J, Janiak F, Danzer C, Ferrari M, Figulla HR (2011). A prospective randomised study using optical coherence tomography to assess endothelial coverage and neointimal proliferation at 6-months after implantation of a coronary everolimus-eluting stent compared with a bare metal stent postdilated with a paclitaxel-eluting balloon (OCTOPUS Trial): rationale, design and methods. EuroIntervention.

[22] Poerner TC, Otto S, Gassdorf J, Nitsche K, Janiak F, Scheller B, Goebel B, Jung C, Figulla HR (2014). Stent coverage and neointimal proliferation in bare metal stents postdilated with a Paclitaxel-eluting balloon versus everolimus-eluting stents: prospective randomized study using optical coherence tomography at 6-month follow-up. Circ Cardiovasc Interv.

[23] Braden B (1986). The Surveyor's Area Formula. Coll Math J.

[24] Meister ALF: Generalia de genesi figurarum planarum et inde pendentibus earum affectionibus. Nov Com Gött 1769, 1(44).

[25] Otto S, Nitsche K, Jung C, Gassdorf J, Janiak F, Goebel B, Figulla HR, Poerner TC (2014). Determinants of neointimal proliferation and stent coverage after intracoronary therapy with drug-eluting devices in stable coronary artery disease: role of endothelial progenitor cells and interleukin-1 family cytokines. J Invasive Cardiol.

[26] Wijns W, Kolh P, Danchin N, Di Mario C, Falk V, Folliguet T, Garg S, Huber K, James S, Knuuti J (2010). Guidelines on myocardial revascularization. Eur Heart J.

[27] Chi J, Hong X, Wang Y, Zhao J, Yang W (2014). Inverse correlation between circulating endothelial progenitor cells with CD34+CD133+ and the severity of coronary atherosclerosis assessed by Syntax score. Am J Med Sci.

[28] Zhang Z, Dong J, Lobe CG, Gong P, Liu J, Liao L (2015). CCR5 facilitates endothelial progenitor cell recruitment and promotes the stabilization of atherosclerotic plaques in ApoE−/− mice. Stem Cell Res Ther.

[29] Liu Y, Hao F, Zhang H, Cao D, Lu X, Li X (2013). Panax notoginseng saponins promote endothelial progenitor cell mobilization and attenuate atherosclerotic lesions in apolipoprotein E knockout mice. Cell Physiol Biochem.

[30] Herlea-Pana O, Yao L, Heuser-Baker J, Wang Q, Wang Q, Georgescu C, Zou MH, Barlic-Dicen J (2015). Chemokine receptors CXCR2 and CX3CR1 differentially regulate functional responses of bone-marrow endothelial progenitors during atherosclerotic plaque regression. Cardiovasc Res.

[31] Keymel S, Kalka C, Rassaf T, Yeghiazarians Y, Kelm M, Heiss C (2008). Impaired endothelial progenitor cell function predicts age-dependent carotid intimal thickening. Basic Res Cardiol.

[32] Moon JH, Chae MK, Kim KJ, Kim HM, Cha BS, Lee HC, Kim YJ, Lee BW (2012). Decreased endothelial progenitor cells and increased serum glycated albumin are independently correlated with plaque-forming carotid artery atherosclerosis in type 2 diabetes patients without documented ischemic disease. Circ J.

[33] Lau KK, Chan YH, Yiu KH, Li SW, Tam S, Lau CP, Kwong YL, Tse HF (2007). Burden of carotid atherosclerosis in patients with stroke: relationships with circulating endothelial progenitor cells and hypertension. J Hum Hypertens.

[34] George J, Afek A, Abashidze A, Shmilovich H, Deutsch V, Kopolovich J, Miller H, Keren G (2005). Transfer of endothelial progenitor and bone marrow cells influences atherosclerotic plaque size and composition in apolipoprotein E knockout mice. Arterioscler Thromb Vasc Biol.

[35] Hagensen MK, Shim J, Thim T, Falk E, Bentzon JF (2010). Circulating endothelial progenitor cells do not contribute to plaque endothelium in murine atherosclerosis. Circulation.

[36] Silvestre JS, Gojova A, Brun V, Potteaux S, Esposito B, Duriez M, Clergue M, Le Ricousse-Roussanne S, Barateau V, Merval R (2003). Transplantation of bone marrow-derived mononuclear cells in ischemic apolipoprotein E-knockout mice accelerates atherosclerosis without altering plaque composition. Circulation.

[37] Peichev M, Naiyer AJ, Pereira D, Zhu Z, Lane WJ, Williams M, Oz MC, Hicklin DJ, Witte L, Moore MA (2000). Expression of VEGFR-2 and AC133 by circulating human CD34(+) cells identifies a population of functional endothelial precursors. Blood.

[38] Haude M, Lee SW, Worthley SG, Silber S, Verheye S, Erbs S, Rosli MA, Botelho R, Meredith I, Sim KH (2013). The REMEDEE trial: a randomized comparison of a combination sirolimus-eluting endothelial progenitor cell capture stent with a paclitaxel-eluting stent. JACC Cardiovasc Interv.

[39] Lim WH, Seo WW, Choe W, Kang CK, Park J, Cho HJ, Kyeong S, Hur J, Yang HM, Cho HJ (2011). Stent coated with antibody against vascular endothelial-cadherin captures endothelial progenitor cells, accelerates re-endothelialization, and reduces neointimal formation. Arterioscler Thromb Vasc Biol.

[40] Pelliccia F, Cianfrocca C, Rosano G, Mercuro G, Speciale G, Pasceri V (2010). Role of endothelial progenitor cells in restenosis and progression of coronary atherosclerosis after percutaneous coronary intervention: a prospective study. JACC Cardiovasc Interv.

[41] Banerjee S, Abu Fadel M, Sarode R, Terada L, Moritz T, Luo P, Hastings J, Brilakis ES, Reda D (2014). Plaque regression and progenitor cell mobilization with intensive lipid elimination regimen (PREMIER) trial design. J Clin Apher.

[42] Kubo T, Xu C, Wang Z, van Ditzhuijzen NS, Bezerra HG (2011). Plaque and thrombus evaluation by optical coherence tomography. Int J Cardiovasc Imaging.

[43] Ino Y, Kubo T, Tanaka A, Kuroi A, Tsujioka H, Ikejima H, Okouchi K, Kashiwagi M, Takarada S, Kitabata H (2011). Difference of culprit lesion morphologies between ST-segment elevation myocardial infarction and non-ST-segment elevation acute coronary syndrome: an optical coherence tomography study. JACC Cardiovasc Interv.

[44] Miyamoto Y, Okura H, Kume T, Kawamoto T, Neishi Y, Hayashida A, Yamada R, Imai K, Saito K, Yoshida K (2011). Plaque characteristics of thin-cap fibroatheroma evaluated by OCT and IVUS. JACC Cardiovasc Imaging.

[45] Kume T, Akasaka T, Kawamoto T, Watanabe N, Toyota E, Neishi Y, Sukmawan R, Sadahira Y, Yoshida K (2006). Assessment of coronary arterial plaque by optical coherence tomography. Am J Cardiol.

[46] Fujii K, Masutani M, Okumura T, Kawasaki D, Akagami T, Ezumi A, Sakoda T, Masuyama T, Ohyanagi M (2008). Frequency and predictor of coronary thin-cap fibroatheroma in patients with acute myocardial infarction and stable angina pectoris a 3-vessel optical coherence tomography study. J Am Coll Cardiol.

[47] Wang L, Parodi G, Maehara A, Valenti R, Migliorini A, Vergara R, Carrabba N, Mintz GS, Antoniucci D (2015). Variable underlying morphology of culprit plaques associated with ST-elevation myocardial infarction: an optical coherence tomography analysis from the SMART trial. Eur Heart J Cardiovasc Imaging.

[48] Yonetsu T, Kato K, Uemura S, Kim BK, Jang Y, Kang SJ, Park SJ, Lee S, Kim SJ, Jia H (2013). Features of coronary plaque in patients with metabolic syndrome and diabetes mellitus assessed by 3-vessel optical coherence tomography. Circ Cardiovasc Imaging.

